# Development and validation of a knowledge, attitude, and practice scale for myopia prevention and control among parents of children and adolescents

**DOI:** 10.3389/fpubh.2026.1904395

**Published:** 2026-08-31

**Authors:** Ming Cong Cao, Guo Xin Bai, Yu Liu, Xue Wang, Yu He Wang

**Affiliations:** 1 Ophthalmology Medical Center, Cangzhou Central Hospital, Cangzhou, China; 2 Department of Optometry, Cangzhou Eye Hospital, Cangzhou, China

**Keywords:** children and adolescents, knowledge, attitude, practice (KAP), myopia prevention and control, parents, scale development

## Abstract

**Objective:**

To develop a knowledge, attitude, and practice (KAP) scale for myopia prevention and control among parents of children and adolescents, and to test its reliability and validity.

**Methods:**

Based on the KAP theory, an initial scale was developed through literature review and expert consultations. From March to April 2026, a convenience sample of 500 parents of children and adolescents from kindergartens, primary schools, and secondary schools was surveyed to test the scale’s psychometric properties. The final scale was formed after reliability and validity analyses.

**Results:**

A total of 477 valid questionnaires were returned. The final scale consists of three dimensions (knowledge, attitude, and practice) with 35 items. The response rates for the two rounds of expert consultation were both 100%, with an authority coefficient of 0.913 and Kendall’s *W* coefficients of 0.372 and 0.413, respectively. The item-level content validity index (I-CVI) ranged from 0.897 to 0.974, and the scale-level content validity index (S-CVI) was 0.945. Exploratory factor analysis extracted three factors explaining 73.52% of the total variance. Confirmatory factor analysis indicated a good model fit (*χ*^2^/d*f* = 1.836, GFI = 0.908, CFI = 0.936, RMSEA = 0.042). The Cronbach’s *α* coefficient for the total scale was 0.944, test–retest reliability was 0.918, and split-half reliability was 0.922.

**Conclusion:**

The developed KAP scale for myopia prevention and control among parents of children and adolescents is well-constructed and exhibits good reliability and validity. It can serve as a valid tool for assessing parents’ KAP levels in this population.

## 1 Introduction

In recent years, the prevalence of myopia among children and adolescents has remained alarmingly high worldwide, posing a major public health challenge (1). The latest surveillance data from China show that the overall myopia rate among children and adolescents is as high as 51.9%, with trends of earlier onset and increasing severity becoming increasingly prominent (2). High myopia significantly increases the risk of irreversible visual impairment, including retinal detachment, macular degeneration, and glaucoma, which can seriously affect the quality of life and future development of affected individuals (3).

The family environment plays a critical role in children‘s visual health, as parents are the primary caregivers who shape daily lifestyle habits and health behaviors. Studies have demonstrated that parents’ knowledge, attitudes, and practices (KAP) regarding myopia prevention are strongly associated with the onset and progression of myopia in their children (4). Parents with better understanding of myopia risk factors are more likely to encourage outdoor activities, monitor screen time, and ensure regular eye examinations—all of which are effective measures for myopia control.

The KAP theory provides a useful framework for understanding health behaviors. It posits that knowledge serves as the foundation for developing positive attitudes, which in turn drive behavioral change (5). This theory has been widely applied in the development of assessment tools across various health domains, including stroke prevention (6), gynecology (7), and psychiatry (8). However, its application to myopia prevention among parents remains limited, despite the clear relevance of parental behaviors to children‘s eye health.

To date, most studies on parental myopia awareness have used self-designed questionnaires without rigorous psychometric validation, making it difficult to compare findings across studies or to reliably assess the effectiveness of health education programs. There is currently no standardized, validated KAP measurement tool specifically designed for Chinese parents of children and adolescents in the context of myopia prevention. Therefore, this study aimed to develop and psychometrically validate a KAP scale for myopia prevention and control among this population, to provide a reliable instrument for assessing parental KAP levels and guiding targeted interventions.

## 2 Materials and methods

This study was a cross-sectional instrument development and validation study. The research was conducted in Cangzhou, China, from January to April 2026, and consisted of four phases: (1) item pool development through literature review and expert consultation; (2) pilot testing for item clarity and feasibility; (3) a cross-sectional survey for psychometric evaluation; and (4) statistical analyses to assess reliability and validity. The study was approved by the hospital ethics committee (Approval No. 2025-029-01), and informed consent was obtained from all participants prior to data collection.

### 2.1 Item pool development

#### 2.1.1 Theoretical framework

The KAP theory was adopted as the conceptual framework. The theory suggests that knowledge is the foundation for developing positive beliefs and correct attitudes, while beliefs and attitudes drive behavioral change. Specifically, knowledge is the basis, attitude is the motivator, and practice is the goal. The three components are interrelated and mutually influential. Accordingly, parents’ knowledge and attitudes toward myopia prevention and control are expected to influence their actual behaviors in managing their children’s myopia.

#### 2.1.2 Literature review

The following Chinese and English keywords were used: “myopia,” “prevention/control,” “children/youth,” “attitude/knowledge/behavior” (and their Chinese equivalents). Searches were conducted in CNKI, Wanfang Data, Chinese Medical Journals Network, VIP Database, PubMed, American Academy of Ophthalmology, International Myopia Institute, and Singapore National Eye Center. The search period covered from database inception to December 2025. A total of 824 articles were initially retrieved. After removing duplicates (EndNote, remaining 657 records), two independent reviewers screened titles, abstracts, and full texts. Disagreements were resolved by discussion or consultation with a third reviewer.

*Inclusion criteria*: ① Studies targeting children and adolescents (aged 3–18 years) or their parents/primary caregivers; ② Studies addressing knowledge, attitudes, beliefs, practices, or influencing factors related to myopia prevention and control; ③ Study types: cross-sectional surveys, cohort studies, intervention studies, scale development/validation studies, systematic reviews, or meta-analyses; ④ Language: Chinese or English; ⑤ Publication type: peer-reviewed journal articles, dissertations, or official institutional reports with complete data. *Exclusion criteria*: ① Studies focusing only on myopia pathogenesis, genetics, animal experiments, or optical parameter design without KAP content; ② Studies with ophthalmologists, optometrists, teachers, or other non-parent populations; ③ Conference abstracts, news reports, popular science articles, commentaries, letters, or reviews without original data; ④ Articles with unavailable full text or incomplete data. After full-text screening, 15 studies were finally included. These were reviewed and used to construct an initial item pool consisting of 33 items across three dimensions.

### 2.2 Expert consultation

From January to February 2026, purposive sampling was used to select 16 experts in myopia prevention and control, pediatric ophthalmology, and ophthalmic nursing for email-based consultation. The consultation questionnaire included instructions, expert background information, an item importance rating form, and questions on familiarity and judgment basis. Expert inclusion criteria: (1) bachelor’s degree or higher; (2) intermediate or higher professional title; (3) ≥5 years of work experience in ophthalmology, ophthalmic nursing, or optometry. Item modification principles: Delete items with coefficient of variation >0.25 or mean importance score <3.5. Revise items based on expert comments and group discussion. Add important missing items identified by experts after group discussion.

### 2.3 Pilot test

A pilot test was conducted in March 2026 among parents of children and adolescents from three kindergartens and schools in Cangzhou. Inclusion/exclusion criteria were the same as for the main survey. Sample size for the pilot test was set at 35, which is consistent with the recommendation by Perneger et al. (9) that a default sample size of 30 participants is adequate for questionnaire pretesting, and the actual number was determined based on practical feasibility. After completing the questionnaire, participants were asked about the clarity of items and any difficulties in understanding. Feedback was collected and reviewed by the research team. No major issues affecting comprehension or response validity were identified, and no modifications were made after the pilot test.

### 2.4 Main survey

#### 2.4.1 Participants

From March to April 2026, a convenience sample of parents of children and adolescents from kindergartens, primary schools, and secondary schools was recruited. Inclusion/exclusion criteria were the same as for the pilot test. Based on exploratory factor analysis requirements (sample size 5–10 times the number of items; final scale had 35 items) and considering 15% invalid responses, 500 parent–child dyads were included. The study was approved by the hospital ethics committee (Approval No. 2025-029-01). Informed consent was obtained from all participants prior to data collection.

#### 2.4.2 Survey instruments

##### 2.4.2.1 General information questionnaire

Collected child’s age, sex, grade, daily near-work time, daily outdoor time on school days and weekends/holidays; parent identity (father/mother), parent age, education level, parent myopia status, weekly accompanying time (excluding sleep).

##### 2.4.2.2 KAP scale for myopia prevention and control among parents

The final scale has three dimensions with 35 items. A 5-point Likert scale was used (1 = strongly disagree/never, 5 = strongly agree/always). Total score ranges from 35 to 175. Of the 35 items, four are reverse-coded (A6, A12, B8, C10). For these items, responses were reversed (1–5, 2–4) before summing scores. Subscale scores were calculated by summing items within each dimension (knowledge: A1–A14, range 14–70; attitude: B1–B9, range 9–45; practice: C1–C12, range 12–60). Higher scores indicate better knowledge, more positive attitudes, or more favorable practices. No item weighting was applied.

#### 2.4.3 Data collection

Team members received standardized training before data collection. The scale was programmed into an electronic survey tool with a QR code. After obtaining informed consent, the questionnaire was distributed via social media or face-to-face scanning. The electronic questionnaire was programmed to require responses to all items before submission, resulting in no missing data in the 477 valid responses.

#### 2.4.4 Statistical analysis

SPSS 26.0 and Jamovi 2.7.18 were used. Normally distributed continuous data were described as mean ± SD; categorical data as frequencies and percentages. Significance level was set at *p* < 0.05. Item analysis: Critical ratio method (CR < 3.00 or non-significant items deleted), correlation coefficient method (items with *r* < 0.4 considered for deletion), and Cronbach’s *α* method (items whose deletion significantly increased overall *α* considered for deletion). Validity analysis: Content validity: I-CVI > 0.78 and S-CVI ≥ 0.90 indicated good content validity. Construct validity: Exploratory factor analysis (EFA) with principal axis factoring as the extraction method and direct oblimin as the oblique rotation; Confirmatory factor analysis (CFA) with fit criteria: *χ*^2^/d*f* < 5, RMSEA < 0.05, CFI > 0.90, GFI > 0.90. Regarding criterion validity, it was not assessed in this study, as there is currently no universally recognized gold-standard KAP questionnaire specifically for parental myopia prevention and control in China, which precluded a direct external comparison. Known-groups validity was assessed by comparing total and subscale scores across key demographic and behavioral variables using independent-sample t-tests and one-way ANOVA. Reliability analysis: Cronbach’s *α*, test–retest reliability, and split-half reliability were calculated. Acceptable values: Cronbach’s *α* > 0.70, split-half reliability >0.80.

## 3 Results

### 3.1 Expert consultation results

#### 3.1.1 Expert characteristics

The 16 experts came from Cangzhou, Shijiazhuang, Urumqi, Beijing, Harbin, Taiyuan, and Longyan. They included 4 males and 12 females; mean age 45.69 ± 4.61 years; work experience: 11–15 years (*n* = 5), 16–20 years (*n* = 4), ≥21 years (*n* = 7); professional titles: intermediate (1, 6.25%), associate senior (10, 62.50%), senior (5, 31.25%); education: bachelor’s (5, 31.25%), master’s (9, 56.25%), doctorate (2, 12.50%); specialties: ophthalmology (14, 87.50%), ophthalmic nursing (1, 6.25%), optometry (1, 6.25%).

#### 3.1.2 Expert positivity, authority, and consensus

The response rate for both rounds was 100%. The authority coefficient was 0.913. Kendall’s *W* coefficients were 0.372 and 0.413 for the two rounds (*p* < 0.05), suggesting acceptable agreement among experts.

#### 3.1.3 Item modifications

##### 3.1.3.1 Round 1

Merged 2 items: “Outdoor activity prevents myopia” + “Balanced diet and adequate sleep prevent myopia” turn to “Outdoor activity, balanced diet, and adequate sleep are effective methods to prevent myopia.” While this item covers multiple behaviors, they were intentionally merged as they collectively represent an overall healthy lifestyle construct for myopia prevention, as recommended by experts.

Added 6 items (e.g., reverse-coded items, items on axial length measurement). Although items on axial length measurement and hyperopia reserve may appear relatively technical, they were retained based on expert consensus, as these concepts are considered core knowledge in contemporary myopia prevention and control and may effectively distinguish between parents with different knowledge levels.

Modified 2 items (e.g., “Wearing glasses does not worsen myopia progression; appropriate or specially designed glasses may slow it down”).

Deleted 2 items due to redundancy.

##### 3.1.3.2 Round 2

No further modifications. Final scale: 3 dimensions, 35 items.

### 3.2 Pilot test results

Thirty-five questionnaires were distributed and all were returned (100% response rate). Mean completion time: 9.27 ± 2.66 min. No further modifications were made.

### 3.3 Main survey results

#### 3.3.1 Participant characteristics

Of the 500 questionnaires distributed, 477 were valid (95.49% response rate). Mean completion time: 8.67 ± 3.62 min. Participant characteristics are shown in Table 1.

**Table 1:** Demographic characteristics of participants (*N* = 477).

Characteristic	Category	*n* (%)
Children/adolescents		
Age (years)	3–6	42 (8.81)
	7–13	210 (44.03)
	14–16	225 (47.17)
Sex	Male	265 (55.56)
	Female	212 (44.44)
Grade	Kindergarten	35 (7.34)
	Primary school	187 (39.20)
	Junior high school	255 (53.46)
Myopia status	Yes	145 (30.40)
	No	332 (69.60)
Daily near-work time	<2 h	145 (30.40)
	2–4 h	190 (39.83)
	>4 h	142 (29.77)
Daily outdoor time (school days)	<1 h	119 (24.95)
	1-2 h	256 (53.67)
	>2 h	102 (21.38)
Daily outdoor time (weekends/holidays)	<1 h	37 (7.76)
	1-2 h	190 (39.83)
	>2 h	250 (52.41)
Parents		
Respondent	Father	100 (20.96)
	Mother	377 (79.04)
Parent age (years)	<30	3 (0.63)
	30–40	302 (63.31)
	41–50	153 (32.08)
	>50	19 (3.98)
Education level	High school or below	273 (57.23)
	Associate/Bachelor’s	170 (35.64)
	Master’s or above	34 (7.13)
Parent myopia	Yes	205 (42.98)
	No	272 (57.02)
Weekly accompanying time (excluding sleep)	<15 h	129 (27.04)
	15–25 h	155 (32.49)
	>25 h	193 (40.46)

#### 3.3.2 Item analysis

Total scale Cronbach’s *α* was 0.944; no item deletion significantly increased this value. All item-total correlations were >0.40, and critical ratios ranged from 4.488 to 10.183 (all >3.00, *p* < 0.05). All 35 items were retained.

#### 3.3.3 Validity

##### 3.3.3.1 Content validity

I-CVI ranged from 0.897 to 0.974, and S-CVI was 0.945, indicating good content validity.

##### 3.3.3.2 Construct validity

*Exploratory factor analysis*: The KMO value was 0.964, and Bartlett’s Test of Sphericity was significant (*χ*^2^ = 8980.932, *p* < 0.001). Data from 355 participants were used for EFA. Three factors with eigenvalues >1 were extracted, explaining 73.52% of the total variance. The factor correlation matrix from the oblique rotation showed moderate correlations among the three factors (knowledge–attitude: *r* = 0.48; knowledge–practice: *r* = 0.52; attitude–practice: *r* = 0.55), supporting the interrelated nature of the KAP dimensions. Factor loadings for all items exceeded 0.6, with no double loadings (all cross-loadings on non-assigned factors were <0.30). The three factors corresponded to knowledge, attitude, and practice (Table 2).

**Table 2:** Factor loadings of the 35-item KAP scale.

Factor	Item no.	Item content	Knowledge loading	Attitude loading	Practice loading
Knowledge (A)	A1	Myopia cannot be cured once it occurs; it can only be controlled.	0.876		
	A2	Outdoor activity, balanced diet, and adequate sleep are effective prevention methods.	0.763		
	A3	Adequate ambient light and correct reading/writing posture help prevent and delay myopia.	0.864		
	A4	Medical myopia control methods include spectacles, contact lenses, medication, and traditional Chinese medicine techniques.	0.732		
	A5	Wearing glasses does not deepen myopia; appropriate or specially designed glasses may slow progression.	0.815		
	A6	Wearing glasses causes eye deformation (reverse-coded).	0.809		
	A7	Frequent eye rubbing, squinting, or tilting the head may be signs of myopia.	0.843		
	A8	Hyperopia reserve is a natural barrier against myopia in children; early depletion leads to early-onset myopia.	0.771		
	A9	Younger age at myopia onset is associated with higher risk of high myopia.	0.858		
	A10	Children of myopic parents have a higher risk of myopia.	0.905		
	A11	High myopia can cause blinding eye diseases such as retinal detachment.	0.817		
	A12	Adult myopia surgery eliminates all eye disease risks, so prevention is unnecessary (reverse-coded).	0.734		
	A13	A refractive development record should be established early to monitor refraction and axial length.	0.828		
	A14	Regular axial length measurement detects myopia risk earlier than visual acuity testing alone.	0.889		
Attitude (B)	B1	Parents are the primary responsible persons for children’s myopia prevention, not just schools or doctors.		0.815	
	B2	Sacrificing some study or leisure time to ensure daily outdoor activity is worthwhile.		0.836	
	B3	I prioritize my child’s sleep duration and balanced diet even when schoolwork is heavy.		0.750	
	B4	I am confident in helping my child develop correct reading/writing posture and eye use habits.		0.823	
	B5	Prevention measures are necessary regardless of whether the child is already myopic.		0.758	
	B6	I worry about my child’s future career limitations and quality of life due to myopia.		0.885	
	B7	I carefully evaluate products or remedies claiming to cure myopia and do not try them easily.		0.838	
	B8	Low myopia does not need glasses; glasses are only needed for high myopia (reverse-coded).		0.826	
	B9	I believe the myopia control methods recommended by professional ophthalmologists are scientific.		0.819	
Practice (C)	C1	Ensure at least 2 h of daytime outdoor activity daily.			0.903
	C2	Limit sugary, fried, and sugary drinks; ensure intake of fresh vegetables, fruits, and high-quality protein.			0.828
	C3	Ensure 8–10 h of sleep per day.			0.813
	C4	Avoid prolonged near work; follow the “20-20-20 rule.”			0.891
	C5	Use both a desk lamp and room ceiling light when reading/writing to avoid insufficient light.			0.765
	C6	Maintain correct reading/writing posture: “one fist, one foot, one inch.”			0.832
	C7	Adjust desk/chair height according to child’s height.			0.806
	C8	Limit non-academic screen time to ≤15 min per session and ≤1 h per day.			0.924
	C9	Prefer large-screen, high-resolution devices; keep distance >40 cm.			0.857
	C10	Playing piano, drawing, or playing with building blocks does not cause myopia (reverse-coded).			0.807
	C11	Establish an eye health record at a professional institution and have professional vision exams every 3–6 months.			0.735
	C12	Actively access eye health education through live streams, campus lectures, etc.			0.728

*Confirmatory factor analysis*: The total sample was randomly split, with 355 participants assigned to EFA and 122 to CFA. CFA was conducted using maximum likelihood estimation with robust standard errors (MLR). The measurement model showed good fit: *χ*^2^/d*f* = 1.836, GFI = 0.908, CFI = 0.936, RMSEA = 0.042, SRMR = 0.046, TLI = 0.931. All standardized factor loadings ranged from 0.51 to 0.88 (all *p* < 0.001). Factor correlations were 0.51 (knowledge–attitude), 0.55 (knowledge–practice), and 0.58 (attitude–practice), supporting the interrelated nature of the three dimensions. Although the CFA sample size (*n* = 122) is relatively modest, the model fit indices and factor loadings all exceeded recommended thresholds, indicating adequate statistical power for model identification. In addition, the EFA and CFA subsamples were comparable in terms of key demographic variables, including child age, sex, grade, and parental education level (all *p* > 0.05), supporting the appropriateness of the random split.

##### 3.3.3.3 Known-groups validity

To assess the scale‘s ability to discriminate between groups expected to differ in KAP levels, we compared total and subscale scores across key demographic and behavioral variables. As shown in Table 3, parents of myopic children had significantly higher total KAP scores than parents of non-myopic children (mean difference = 13.10, 95% CI: 9.97–16.23, *p* < 0.001). Higher parental education levels were associated with higher total scores (*p*-for trend <0.001). Parents who reported longer daily outdoor time on school days had higher practice subscale scores (*p* < 0.001). These results support the known-groups validity of the scale.

**Table 3:** Known-groups validity of the KAP scale.

Variable	Category	*n*	KAP total score (mean ± SD)	Mean difference [95% CI]	*p*-value
Children‘s myopia status	Myopic	145	143.65 ± 16.54	13.10 [9.97, 16.23]	<0.001
	Non-myopic	332	130.55 ± 15.78	Reference	
Parental education level	High school or below	273	129.68 ± 16.37	Reference	<0.001
	Associate/Bachelor’s	170	141.24 ± 16.08	11.56 [8.28, 14.84]	
	Master’s or above	34	148.56 ± 16.30	18.88 [12.30, 25.46]	
Daily outdoor time (school days)	<1 h	119	31.73 ± 4.48	Reference	<0.001
(Practice subscale score)	1-2 h	256	34.65 ± 4.38	2.92 [1.78, 4.06]	
	>2 h	102	36.81 ± 4.21	5.08 [3.56, 6.60]	

#### 3.3.4 Reliability

The total scale Cronbach’s *α* was 0.944; the knowledge subscale *α* was 0.876, the attitude subscale *α* was 0.838, and the practice subscale *α* was 0.909. To assess test–retest reliability, the 35 participants from the pilot sample completed the questionnaire again 2 weeks after the initial survey. The intraclass correlation coefficient (ICC) with a two-way random-effects model for absolute agreement was used. The ICC values were 0.889 (95% CI: 0.78–0.95) for the knowledge subscale, 0.924 (95% CI: 0.86–0.96) for the attitude subscale, 0.909 (95% CI: 0.83–0.95) for the practice subscale, and 0.918 (95% CI: 0.85–0.96) for the total scale. Split-half reliability: total = 0.922 (knowledge = 0.917, attitude = 0.942, practice = 0.934).

#### 3.3.5 Subscale descriptive statistics

The mean scores for the three subscales were as follows: knowledge dimension: (54.37 ± 7.82) points; attitude dimension: (38.52 ± 4.96) points; practice dimension: 42.86 ± 6.53 points. The total scale mean was (135.75 ± 15.89) points.

Floor and ceiling effects were minimal. No respondents scored at the theoretical minimum on any subscale or the total scale. The percentage of respondents scoring at the theoretical maximum was 0.21% for the total scale (*n* = 1), 0.42% for the knowledge subscale (*n* = 2), 4.82% for the attitude subscale (*n* = 23), and 0.84% for the practice subscale (*n* = 4). These results indicate that the scale has adequate range and no substantial floor or ceiling effects.

## 4 Discussion

### 4.1 Scientific soundness of the scale

This scale was developed based on the KAP theory, combined with a systematic literature review and two rounds of expert consultation. The 16 experts came from multiple provinces and specialties closely related to myopia prevention (ophthalmology, optometry, ophthalmic nursing). This geographic and disciplinary diversity helped ensure that the item pool reflected a broad range of perspectives and reduced the potential for regional biases in item evaluation, thereby strengthening the content validity of the scale. The authority coefficient was 0.913, and the experts’ positive coefficient was 100%. Kendall’s *W* values indicated good consensus. The validity and reliability indices all exceeded the recommended thresholds, indicating that the scale possesses strong psychometric properties. The cumulative variance explained in the present study is comparable to that reported in other KAP scale development studies in health domains (10). The CFA fit indices met or exceeded conventional benchmarks, indicating a well-fitting model. Collectively, these findings suggest that the scale can reliably capture parental KAP levels across the three theoretically defined dimensions. Overall, the scale demonstrates strong scientific rigor and psychometric robustness.

Regarding the relatively high Cronbach’s *α* for the total scale, we acknowledge that this may partly reflect some degree of item redundancy. While some researchers have noted that excessively high *α* values may suggest item redundancy, others have argued that this is not necessarily problematic in multidimensional scales if the factor structure remains theoretically coherent (11). In this study, several observations suggest that the high α does not compromise the scale’s validity: First, the three-factor structure was confirmed by EFA and CFA, indicating that the items load onto distinct yet correlated dimensions rather than a single latent construct (12). Second, the inter-factor correlations (*r* = 0.48–0.58) were moderate, supporting the discriminant validity of the subscales despite the high total-scale *α* (13). Third, the scale was designed to cover the full breadth of KAP concepts related to myopia prevention, and retaining all 35 items was necessary to ensure content coverage. Nevertheless, future studies may consider shortening the scale by removing redundant items while maintaining its psychometric integrity.

### 4.2 Practical utility

Compared with previous studies that relied on locally developed, non-standardized questionnaires (14–18), our validated scale offers a standardized framework for assessing parental KAP across diverse populations. Our findings—showing a notable gap between high attitude scores and lower practice scores—align with those of Wang et al. (14) and Zhan et al. (15), who also reported favorable attitudes alongside suboptimal practices. However, unlike their unvalidated instruments, our scale provides quantified reliability and validity estimates, enabling more robust cross-study comparisons. This approach facilitates comparable assessments, supports robust cross-study syntheses, and offers substantial practical utility for designing targeted interventions. Consequently, the KAP scale developed in the present study provides a standardized instrument that can be consistently applied by different researchers across diverse populations and at various time points. This approach facilitates comparable assessments, supports robust cross-study syntheses, and offers substantial practical utility for designing targeted interventions. Reverse-coded items were included in the knowledge and attitude dimensions to minimize acquiescence bias, i.e., the tendency of respondents to agree with all statements regardless of content. Such items are commonly used in KAP scales to encourage more careful reading and thoughtful responses (19). No significant comprehension issues were identified during the pilot test, supporting the clarity and appropriateness of the reverse-coded items.

Beyond its value as a standardized instrument, the findings of this study offer several insights into parental KAP regarding myopia prevention. We observed that parents had relatively high attitude scores but low practice scores, suggesting that positive attitudes alone may not translate into effective practices without addressing practical barriers such as time constraints, competing academic priorities, or limited access to eye care resources. Additionally, parents of myopic children demonstrated higher knowledge scores, possibly reflecting a “teachable moment” effect in which having a child diagnosed with myopia motivates parents to actively seek relevant information. The scale has practical utility across multiple settings. Clinically, it can help ophthalmologists and primary care providers identify parents with inadequate knowledge or unfavorable practices, facilitating individualized health education. At the community and school levels, it can serve as a standardized outcome measure for evaluating the effectiveness of myopia prevention programs. At the policy level, the scale may assist health authorities in monitoring trends in parental KAP over time and assessing the impact of national public health campaigns aimed at reducing childhood myopia. Collectively, these applications support the translation of the scale into routine practice to guide targeted, evidence-based interventions.

### 4.3 Evidence relevance

Beyond its psychometric properties, the content of the three dimensions is also grounded in current evidence on childhood myopia prevention. The knowledge items address established risk factors for myopia, including parental myopia, outdoor time, near-work load, and axial length monitoring (20). The attitude items assess parents’ perceived responsibility, willingness to balance lifestyle adjustments against academic pressures, and trust in professional advice—key drivers of behavior change within the KAP framework (21, 22). The practice items cover specific behavioral recommendations derived from authoritative national guidelines. For example, the recommendation for at least 2 h of daily outdoor activity (Item C1), the 20-20-20 rule (Item C4), and screen time limits (Items C8-C9) are sourced from the Core Knowledge Ten Points for Myopia Prevention and Control in Children and Adolescents (23). Similarly, items on hyperopia reserve monitoring (Item A8) and regular axial length measurement (Items A13–A14) are grounded in the Guidelines for Myopia Prevention and Control (2024 Edition) (24) and the Expert Consensus on the Application of Axial Length in Myopia Prevention and Control Management (21), which recommend regular assessment of these parameters for early myopia risk detection. These evidence-informed components support the scale’s utility in reflecting modifiable factors relevant to current myopia prevention strategies (25). The relevance of these practice items is further supported by recent international evidence demonstrating that educational models with higher near-work demands are associated with faster myopia progression in children (26).

## 5 Limitations and future directions

This study has several limitations. First, the sample was drawn from a single city (Cangzhou) through convenience sampling, which may limit generalizability. Parental awareness and practices regarding myopia may vary by region and socioeconomic context, and our sample may have overrepresented more health-conscious parents. Therefore, we plan to conduct external validation in diverse geographic and socioeconomic settings before promoting broader application of the scale. Second, convenience sampling via an online survey may have introduced selection bias, as concerned parents were more likely to participate. In addition, mothers were overrepresented, which may bias results toward maternal perspectives. Future studies should include more balanced samples through offline recruitment and targeted enrollment of fathers to enhance generalizability. Third, the cross-sectional design does not allow assessment of test–retest reliability over longer intervals. In addition, measurement invariance across parental sex was not examined due to the limited sample size of fathers (*n* = 100), which precluded reliable multi-group CFA. Future studies with larger and more balanced samples should assess this property. Fourth, criterion validity was not examined due to the absence of a suitable external criterion, as noted in the Methods section. Fifth, although the scale is intended for use in future health promotion programs, its sensitivity to change has not yet been empirically tested. Future studies should assess responsiveness through longitudinal intervention designs to confirm its utility in evaluating program effectiveness. Finally, social desirability bias may have influenced self-reported practices. Future research should examine the scale’s sensitivity to change following interventions, and its predictive validity for child myopia outcomes. The scale may also be adapted for use in community health settings to identify at-risk families and guide health education.

## 6 Conclusion

The developed knowledge, attitude, and practice scale for myopia prevention and control among parents of children and adolescents is well-constructed and exhibits good reliability and validity. It can serve as an effective tool for assessing parents’ KAP levels and for evaluating the impact of health promotion programs in this area.

## Data Availability

The original contributions presented in the study are included in the article/supplementary material, further inquiries can be directed to the corresponding author.
